# 

**Published:** 1904-02-15

**Authors:** 


					﻿THE
DENTAL REGISTER
EDITED BY
NELV1LLE S. HOFF, D. D.S.,
ANN ARBOR, MICH.
Vçoip LyıfL . J, February 15, 1004.	No. 2.
PRICE, ONE DOLLAR A YEAR IN ADVANCE.
PUBLISHED MONTHLY BY
SAMUEL A, CROCKER & CO.,
THE OHIO DENTAL AND SURGICAL DEPOT.
to Í5O W. “tli St., Ciiieiiintili. O.
Entered at the Postoffice at Cincinnati, 0., as second-class mail matter.
				

